# PI3K Orchestrates T Follicular Helper Cell Differentiation in a Context Dependent Manner: Implications for Autoimmunity

**DOI:** 10.3389/fimmu.2018.03079

**Published:** 2019-01-07

**Authors:** Silvia Preite, Bonnie Huang, Jennifer L. Cannons, Dorian B. McGavern, Pamela L. Schwartzberg

**Affiliations:** ^1^National Institute of Allergy and Infectious Disease, National Institutes of Health, Bethesda, MD, United States; ^2^National Human Genome Research Institute, National Institutes of Health, Bethesda, MD, United States; ^3^National Institute of Neurological Disorders and Stroke, National Institutes of Health, Bethesda, MD, United States

**Keywords:** Tfh cells, Tfr, ICOS, PI3K, APDS, PASLI, autoimmunity

## Abstract

T follicular helper (Tfh) cells are a specialized population of CD4^+^ T cells that provide help to B cells for the formation and maintenance germinal centers, and the production of high affinity class-switched antibodies, long-lived plasma cells, and memory B cells. As such, Tfh cells are essential for the generation of successful long-term humoral immunity and memory responses to vaccination and infection. Conversely, overproduction of Tfh cells has been associated with the generation of autoantibodies and autoimmunity. Data from gene-targeted mice, pharmacological inhibitors, as well as studies of human and mice expressing activating mutants have revealed that PI3Kδ is a key regulator of Tfh cell differentiation, acting downstream of ICOS to facilitate inactivation of FOXO1, repression of *Klf2* and induction of *Bcl6*. Nonetheless, here we show that after acute LCMV infection, WT and activated-PI3Kδ mice (*Pik3cd*^E1020K/+^) show comparable ratios of Tfh:Th1 viral specific CD4^+^ T cells, despite higher polyclonal Tfh cells in *Pik3cd*^E1020K/+^ mice. Thus, the idea that PI3K activity primarily drives Tfh cell differentiation may be an oversimplification and PI3K-mediated pathways are likely to integrate multiple signals to promote distinct effector T cell lineages. The consequences of dysregulated Tfh cell generation will be discussed in the context of the human primary immunodeficiency “Activated PI3K-delta Syndrome” (APDS), also known as “p110 delta-activating mutation causing senescent T cells, lymphadenopathy and immunodeficiency” (PASLI). Overall, these data underscore a major role for PI3K signaling in the orchestration of T lymphocyte responses.

## Introduction

Naïve CD4^+^ T helper (Th) cells play pivotal roles in adaptive immunity through the differentiation into distinct cytokine-producing effector subsets that specifically fight a wide range of pathogens and tumors (1). T follicular helper (Tfh) cells provide help to B cells for the formation of germinal centers (GCs) (2–4), a specialized microenvironment where clonal expansion of B cells, immunoglobulin diversification, affinity maturation, and development of memory B and long-lived plasma cells occur in response to immune challenge (5). The outcome of GC reactions requires proper help provided by Tfh cells (6–9). Most successful human vaccines are based on the generation of long-term protective humoral responses derived from the interactions of Tfh and GC B cells; however, Tfh cells can also promote dysregulated responses and autoimmunity (7, 10, 11). It is, therefore, critical to understand factors that promote or limit Tfh cells to elicit tightly controlled GC responses.

Recent data from gene-targeted mice, as well as mice and humans expressing activating mutants of phosphatidylinositol 3-kinase delta (PI3Kδ), suggest that PI3K activity is an essential component of pathways driving Tfh cell and GC formation (12–16). In this review, we discuss PI3Kδ-mediated pathways involved in the generation, maintenance and function of Tfh cells, including cellular receptors that activate PI3K within T cells, molecular pathways activated, and implications for autoimmunity, with a focus on the genetic disease APDS/PASLI.

## PI3K Signaling in Immunity

### PI3K Signaling

**Figure 1:**
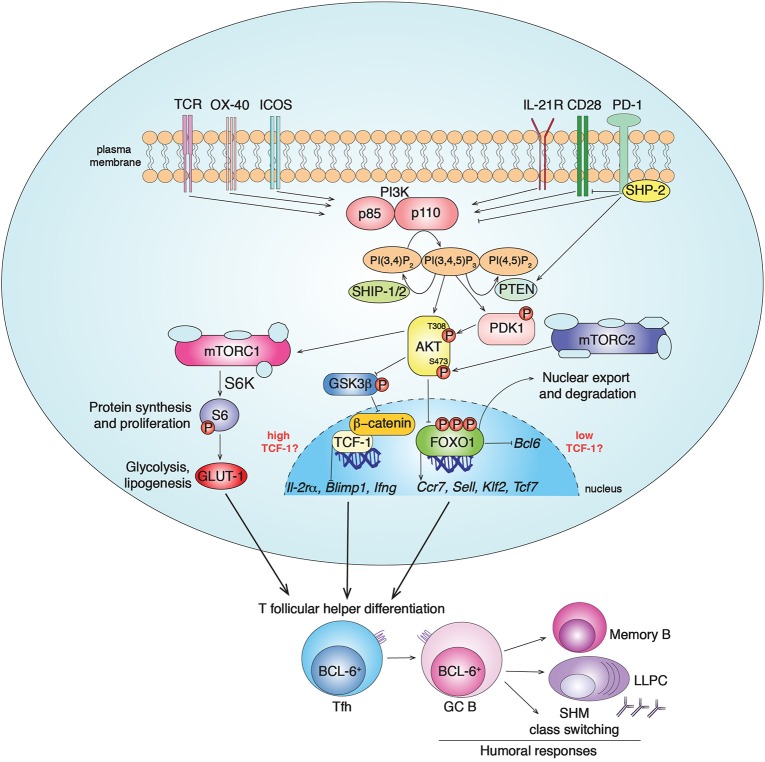
PI3K signaling pathways in Tfh cell differentiation. Class IA PI3Ks (PI3Kα, PI3Kβ, PI3Kδ) are lipid kinases composed by a regulatory (p85) and a catalytic (p110) subunit. Multiple receptors activate PI3K in CD4^+^ T cells, including TCR, CD28, ICOS, OX-40 and IL-21R, leading to PI3K recruitment to the plasma membrane and conversion of the membrane lipid PI(4,5)P_2_ to PI(3,4,5)P_3_. In T cells, chemokine receptors, including CXCR5, preferentially drive the activation of the class IB PI3Kγ (19). PI3K activity is counteracted by the inhibitor receptor PD-1 that blocks CD28 signal transduction through SHP-2 recruitment and PTEN induction. The phosphatases PTEN and SHIP-1/2 counteract PI3K signaling by converting PIP_3_ to PI(4,5)P_2_ and PI(3,4)P_2_, respectively. PIP_3_ recruits to the plasma membrane proteins containing pleckstrin homology domains, such as AKT and PDK1. The serine/threonine kinase AKT gets activated by phosphorylation by PDK1 (at Thr308) and mTORC2 (at Ser473). In turn, activated pAKT phosphorylates inhibitors of mTORC1 leading to its activation. mTORC1 phosphorylates several factors including S6-kinase (S6K), that phosphorylates S6, driving protein synthesis and cell proliferation, important events for Tfh cell differentiation. pAKT also phosphorylates the FOXO-1 transcription factor leading to its export outside the nucleus and degradation by binding 14-3-3 proteins. FOXO-1 represses *Bcl6*, essential for Tfh cells, and promotes expression of multiple genes including *Sell, Tcf7, Ccr7*, and *Klf2*. While KLF-2 restrains Tfh cell program through multiple mechanisms, TCF-1 promotes Tfh cell formation by inhibiting *Il-2r*α, *Blimp1, Ifng*. At the same time, it has been shown that mTORC2-pAKT may also support TCF-1 activity through the inactivation of GSK3β, an inhibitor of β-catenin and TCF-1. Overall, PI3K pathways drive BCL-6^+^ Tfh cell differentiation that coordinates GC responses and humoral immunity after infections and vaccination through the generation of memory B cells and long-lived plasma cells (LLPC).

The PI3Ks are a family of heterodimeric lipid kinases that are activated downstream of a variety of receptors, including growth factor, antigen, costimulatory, cytokine, chemokine, and Toll-like receptors (17, 18). Class IA PI3Ks consist of a p85 regulatory and a p110 catalytic subunit that catalyzes the addition of a phosphate to the membrane phospholipid PI(4,5)P_2_, to generate phosphoinositide 3,4,5-triphosphate (PIP_3_). PIP_3_ helps recruit signaling molecules containing pleckstrin homology and other PIP_3_-binding domains to the plasma membrane to propagate signaling cascades (Figure 1). Mammals express three class IA catalytic isoforms: the broadly expressed p110α and p110β, and p110δ, which is expressed primarily by immune cells (17). Notably, PI3Kδ is activated by a variety of cell-surface receptors that are critical for Tfh cell differentiation, localization and function, including the T-cell receptor, CD28, and ICOS co-receptors, and cytokine receptors (17).

Activated-PI3K coordinates the recruitment of molecules such as PDK1 that phosphorylates and activates the serine/threonine kinase AKT, which in turn phosphorylates multiple targets. Among these are the FOXO transcription factors, which are then sequestrated outside the nucleus by 14-3-3 proteins and degraded. FOXOs regulate transcription of multiple genes involved in lymphocyte development, differentiation and function (17, 20). Another downstream effector of PI3K is the mammalian Target of Rapamycin kinase (mTOR), which forms two complexes, mTORC1 and mTORC2, with different scaffolding partners (21). AKT activates mTORC1, an ancient regulator of metabolism, protein synthesis, and cell growth. mTORC2 is essential to fully phosphorylate and activate AKT, thus contributing to downstream signaling, including FOXO1 inactivation, and actin reorganization (21–23). PI3K is counteracted by the lipid-phosphatases PTEN and SHIP-1/2 that convert PIP_3_ to PI(4,5)P_2_ and PI(3,4)P_2_, respectively, (17) (Figure 1).

The importance of PI3Kδ in lymphocyte function is highlighted by the human primary immunodeficiency APDS/PASLI, in which patients are heterozygous for activating mutations in *PIK3CD*, the gene encoding p110δ. These patients show immunodeficiency and lymphopenia, as well as lymphoproliferation and autoimmunity (14, 15, 24, 25). Four independent groups, including us, have recently generated mouse models expressing the E1020K activating mutant of p110δ, which recapitulate many features of APDS/PASLI (16, 26–28). Notably, patients and *Pik3cd*^E1020K/+^ mice exhibit elevated circulating Tfh cells and GCs associated with autoantibody production (15, 16, 24, 29). Mice that express constitutively active p110α in T cells (30) or have a T cell-specific deletion of PTEN (13) also have elevated Tfh cell frequencies, supporting a more general connection between PI3K activity and Tfh cells. Nonetheless, the observation that p110δ-inactivation in T cells abrogates Tfh cell generation, supports a non-redundant role of p110δ in this process (12, 13). Together, these data provide strong evidence that PI3Kδ is an important component of pathways driving Tfh cell differentiation.

### Tfh Cell Differentiation

The generation of Tfh cells is a multistage process that requires the integration of signals from different cell types (31). In the T cell zone of secondary lymphoid organs, antigen-presenting dendritic cells (DCs) activate T cells to initiate the pre-Tfh cell program, leading to induction of the costimulatory molecule ICOS and chemokine receptor CXCR5, as well as downregulation of CCR7, which together permit migration to the T-B cell border zone (32–34). Here, activated B cells receive signals from pre-Tfh cells to differentiate either along extra-follicular or GC pathways (5, 35). Cognate interactions with activated B cells help promote the differentiation into GC-Tfh cells (36), identified as CXCR5^hi^PD-1^hi^Foxp3^−^CD4^+^ T cells that also express high levels of ICOS, CD40L, and the Tfh-master transcription factor BCL-6, which are all critical for Tfh cell differentiation (37, 38). In turn, Tfh cells provide signals via costimulatory molecules and cytokines that help establish and maintain GCs. Thus, the generation of Tfh cells and GC reactions requires intimate communication between T and B cells involving multiple receptors that activate PI3K.

### ICOS-PI3K Pathways in Tfh Cells

One of the key costimulatory receptors expressed by Tfh cells is ICOS, a CD28 family member. CD28 and ICOS both activate PI3Kδ and are required for Tfh cell development and function. CD28-CD80/CD86 interactions are involved in early T cell activation, including initial induction of ICOS, BCL-6, and CXCR5 (39), which are necessary for Tfh cell formation; *Cd28*^−/−^ mice show a total absence of Tfh cells and thymus-dependent (TD) germinal centers (40–42). ICOS is upregulated on activated T cells shortly after TCR stimulation and interacts with ICOS-ligand (ICOS-L) on antigen presenting cells including DCs and B cells (43). *Icos*^−/−^ and *Icosl*^−/−^ mice display severely reduced humoral response to TD-antigens characterized by a lack of immunological memory and defective GC formation (44–49). Patients lacking ICOS display a common variable immunodeficiency (CVID) with reduced circulating Tfh cells (50, 51). Conversely, mutations affecting Roquin, a negative post-transcriptional regulator of *ICOS* mRNA, increase Tfh cells and drive autoimmunity (52, 53).

ICOS helps drive multiple stages of Tfh cell differentiation, including the early generation of CXCR5^high^ T cells, modulation of other chemokine and homing receptors through regulation of the KLF2 transcription factor (39, 54), and T:B cell non-cognate interactions that promote T cell motility at the T:B cell border (55). ICOS-ICOS-L interactions are also critical for localization and maintenance of GC-Tfh cells (9, 39, 54).

The essential role of PI3K in ICOS function was highlighted by data showing that mutation of the p85-binding site, which selectively abrogates PI3K recruitment, led to defects in Tfh cell formation similar to ICOS-deficiency (56). Inhibition of p110δ also prevented ICOS-mediated changes in cell migration and morphology *in vitro* (55). Conversely, we found that activated-PI3Kδ mice show T cell-intrinsic increases in Tfh cell differentiation, even in the presence of blocking anti-ICOS-L antibody, therefore bypassing the requirement for ICOS for Tfh cell development (16). Thus, PI3K appears to be a major effector of ICOS, required for Tfh cell formation and maintenance.

### PI3K Signaling Downstream of ICOS

After ICOS ligation, activated-PI3Kδ transduces its signals through several intermediates, including pAKT-mediated inactivation of FOXO1 (20). FOXO1 transcriptionally represses *Bcl6*, a driver of Tfh cell differentiation (30, 57, 58); strong PI3K activity relieves this repression. FOXO1 also transcriptionally activates *Klf2* (59), which restrains Tfh cells and promotes alternative T helper subsets through at least four mechanisms: (1) induction of *S1pr*1, downregulation of which is essential for Tfh cell retention in GC and efficient polarization; (2) induction of BLIMP1, which negatively regulates *Bcl6* and Tfh cell generation; (3) induction of T-bet and GATA3 which drives Th1 and Th2 cell differentiation, respectively; and (4) repression of *Cxcr5* (39, 60). Accordingly, *Foxo1*^−/−^ CD4^+^ T cells generate increased percentages of pre-Tfh cells (CXCR5^int^BCL-6^int^) early post-immunization, even in the presence of anti-ICOS-L (57), similar to cells expressing activated-PI3Kδ (16). These data support the ICOS-PI3Kδ-FOXO1 pathway as critical for Tfh cell development; accordingly, *Pik3cd*^E1020K/+^ CD4^+^ T cells exhibit elevated pFOXO1 upon TCR stimulation, even without further ICOS re-stimulation (16). Furthermore, an AKT-resistant mutant of FOXO1 prevents increased Tfh cells in the presence of activated-PI3Kδ (16). It is also of note that ICOS is a stronger inducer of PI3K than CD28, resulting in greater inhibition of FOXO1; this may account for the inability of CD28 to compensate for ICOS-deficiency in promoting Tfh cells (39, 42, 56, 61, 62). However, *Foxo1*^−/−^ T cells show defective GC-Tfh (CXCR5^high^BCL-6^high^) cell formation (57) which is not observed with activated-PI3Kδ (16). Thus, cells expressing activated-PI3Kd likely still retain some FOXO1 activity. FOXO1 is required for sustained surface ICOS expression (57), providing a possible explanation for this defect. Indeed, chromatin immunoprecipitation and deep sequencing revealed FOXO1 binding sites in multiple genes that influence Tfh cell fate, including *Cxcr4, Batf, Irf4, Icos*, and *Prdm1* (57, 63).

Nonetheless, despite increased GC-Tfh cell differentiation, *Pik3cd*^E1020K/+^ mice show disorganized GCs with increased Tfh cell infiltration and impaired class-switched antigen-specific responses to immunization (16, 27, 28). Multiple factors may contribute to these poor responses, including impaired B cell selection due to increased Tfh cells (7) and Tfh cell mis-localization (16), or intrinsic B cell defects. Indeed, although deletion of p110δ in B cells only minimally affected GC formation and T cell-dependent humoral responses after protein immunization (13), activated-PI3Kδ drove B cell-intrinsic increases in GC B and plasma cells, as well as impaired class-switched antibody production (16, 28). Increased GC B cells may in turn further drive expanded Tfh cell numbers, contributing to immune dysregulation. Additionally, increased GCs that fill the follicular dendritic cell network at baseline, may prevent new GC formation as mice age (16). Whether and how FOXO1 contributes to defects in antigen-specific responses or whether additional downstream effectors of PI3K are involved remain intriguing questions. It should also be noted that additional receptors expressed by Tfh cells, including OX-40, and IL-21R activate PI3K (64) and may contribute to expanded Tfh cell populations in these mice; in contrast, PD-1, an inhibitory receptor highly expressed by Tfh cells (65), counteracts PI3K by blocking CD28 signaling and increasing PTEN expression (66–69) (Figure 1).

## Context-Dependent Roles For PI3K in T Cell Differentiation

### Viral Infection

**Figure 2:**
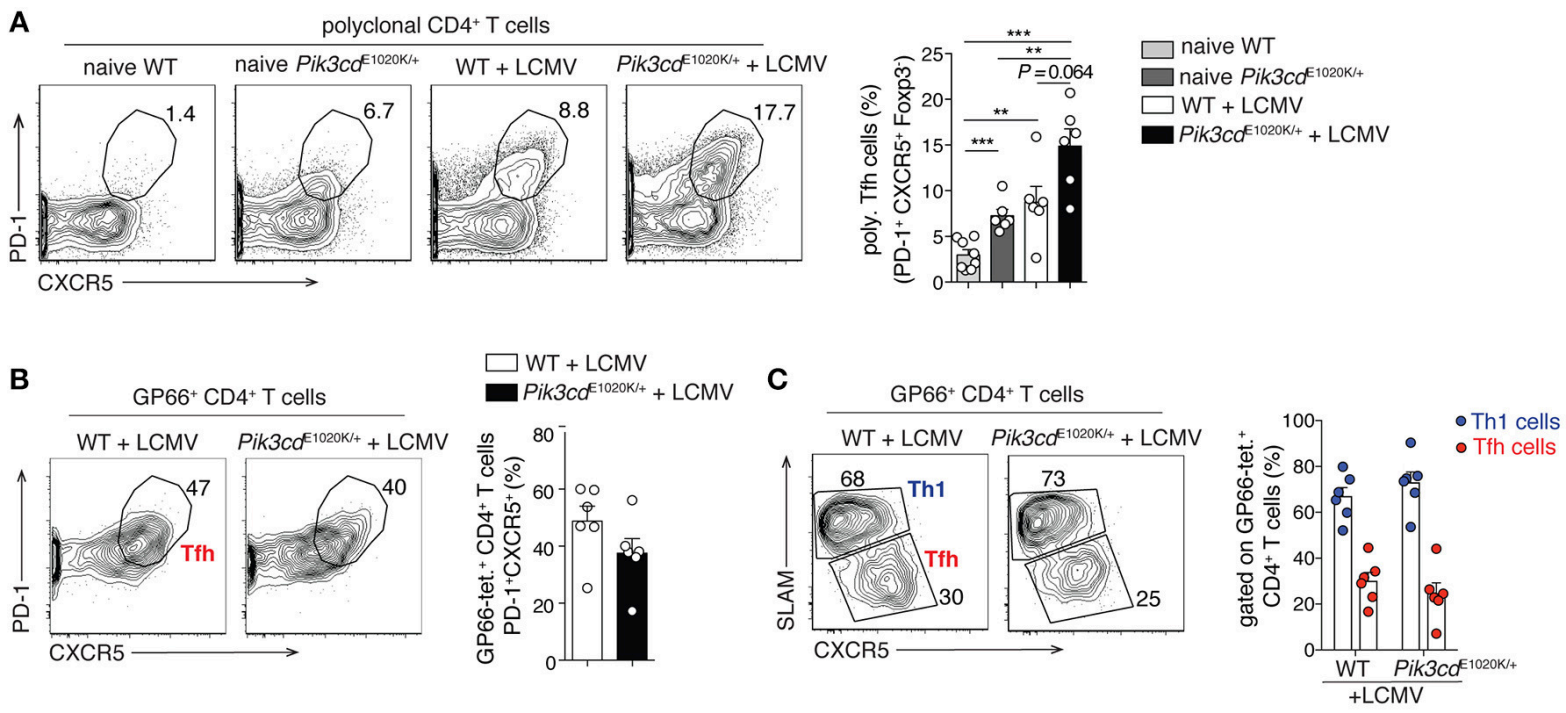
LCMV infection promotes comparable Th1/Tfh cell differentiation within GP66-tetramer^+^ T cells, despite increased polyclonal Tfh cells in *Pik3cd*^E1020K/+^ mice. 2/3-month-old WT and *Pik3cd*^E1020K/+^ mice were infected i.v. with LCMV Arm and analyzed in the spleen 7/8 days post infection. Naïve WT and *Pik3cd*^E1020K/+^ mice were analyzed as control. **(A)** Representative contour plots and summary histogram of polyclonal (GP66 tetramer negative) PD-1^+^CXCR5^+^Foxp3^−^ Tfh cells (percentage of CD4^+^B220^−^GP66^−^ T cells). **(B)** Representative contour plot and summary histogram of Tfh cells (PD-1^+^CXCR5^+^), gated on GP66-tetramer^+^ CD4^+^B220^−^Foxp3^−^ T cells. **(C)** Representative contour plot and summary histogram of Th1 (SLAM^hi^CXCR5^lo^) and Tfh (SLAM^lo^CXCR5^hi^) cells (gated on GP66-tetramer^+^CD4^+^B220^−^Foxp3^−^ T cells). We were unable to evaluate GP66^+^ Tfr cells due to the low numbers of GP66^+^ Foxp3^+^ cells (70). However, polyclonal Tfr cells were increased after LCMV infection (data not shown), as we have previously reported in naïve mice (16). **(A–C)**, *n* = 5–8. Data are representative of three independent experiments and are expressed as mean ± SEM with each dot indicating one mouse. Significance analyzed by Mann-Whitney *U-*test. ** *p* < 0.01; *** *p* < 0.001.

Although the connection between ICOS and PI3Kδ provides strong evidence for PI3Kδ driving Tfh cells, the view that PI3K exclusively promotes Tfh cells may be a simplification; this is particularly apparent when looking at the differentiation of Tfh vs. Th1 cells during viral infection. In response to viral or strong Th1 polarizing infections, CD4^+^ T cells undergo an early bifurcation such that up to 50% of viral-specific T cells express BCL-6 and become Tfh cells, while the rest express BLIMP1 and SLAM, and become IFNγ-producing Th1 cells (54). Although activated-PI3Kδ increased percentages of Tfh cells at baseline and in response to immunization (16), as well as polyclonal CXCR5^+^PD-1^+^ Tfh cells after LCMV infection (Figure 2A), it did not alter Tfh cell percentages, nor Tfh/Th1 ratios, within viral-specific GP66-tetramer^+^ CD4^+^ T cells in the same mice (Figures 2B,C). We also observed increased percentages of circulating CXCR3^+^ Tfh1 cells in patients with APDS/PASLI compared to controls (16), suggesting that PI3K can drive both Tfh cells and Type 1 immunity. Thus, PI3K activity may promote multiple effector T cell lineages and the effects of PI3K on Tfh cells may depend on the activating stimuli and microenvironment.

### IL2 Signaling

Among potential PI3K-mediated signaling pathways that influence Tfh and Th1 cell differentiation are those downstream from the cytokine IL-2. Early data suggested that PI3K is activated by the IL-2R signaling complex (71–73); PI3K inhibitors arrest IL-2 induced CTL growth (74, 75). However, recent reports question the direct connection between IL-2 and PI3K activation (76), as that: (1) certain PI3K inhibitors (such as LY294002) have off-target effects (77); (2) many studies evaluate pAKT^S473^ and pS6, rather than pAKT^T308^, which more accurately reflects PI3K activity (78); and (3) IL-2 can promote mTORC1 activation independent of PI3K (79). Indeed, IL-2 potently inhibits Tfh cell generation via STAT5-mediated induction of BLIMP1 (80–82); BLIMP1^+^ Th1 cells express high levels of the high-affinity IL-2 receptor, CD25, and pSTAT5. As that IL-2 activates multiple signaling pathways, the integration, kinetics, and balance of these and other signals elicited in response to multiple receptors, may ultimately help determine T helper cell fates.

### Metabolic Pathways in Tfh vs. Th1 Cells

Other PI3K-mediated signaling pathways that may influence both Tfh and Th1 cells are those involving mTORC1 and mTORC2. During acute LCMV infection, Th1 cells appear more proliferative and bio-energetically demanding with greater glucose metabolism and metabolic respiration than Tfh cells (83). Data suggest that these Th1 cells were more dependent on the IL-2-PI3K-AKT-mTORC1 axis, which preferentially promoted BLIMP1^+^ Th1 cells at the expense of BCL-6^+^ Tfh cells and humoral immunity (83, 84). However, other studies have demonstrated requirements for mTORC1 and mTORC2 in driving Tfh cells in Peyer's Patches at steady state and in the periphery after LCMV infection and immunization (30, 85). Mechanistically, Tfh cells were supported by mTORC1-promotion of pS6, GLUT1 expression, glycolysis, lipogenesis and overall proliferation; and by mTORC2-pAKT, which decreased FOXO1 activity (30).

While these studies provide conflicting conclusions on the requirements for PI3K and downstream effectors for Tfh cells, this may result from different experimental systems (knockdown vs. knockout) as well as bio-energetic demands during immune challenges. However, there is also evidence that mTOR may be activated independently of PI3K via pathways involving nutrient sensing that may also affect T helper cell differentiation (22, 79, 86, 87).

### PI3K-TCF-1 Cross-Talk

Several recent studies revealed that the transcription factor TCF-1 is expressed at high levels in Tfh cells after viral infection and plays an essential role in their generation and maintenance, via repression of *Il2ra*, and *Prdm1* (which encodes BLIMP1), promotion of *Bcl6* (55, 88–90), and possibly repression of *Ifng* (91). Intriguingly, PI3K has been implicated both positively and negatively in TCF-1 regulation (92, 93). In CD8^+^ T cells, *Tcf7*, which encodes for TCF-1, is induced by FOXO1 (94), and both are required for memory T cell formation (95–98). Strong PI3K signaling would therefore be expected to decrease TCF-1 levels (25), as observed in studies of asymmetric cell division (92, 93). Conversely, a positive link between PI3K/AKT and TCF-1 has been proposed via β-catenin (85), a coactivator of TCF-1 that is negatively regulated by phosphorylation by Glycogen Synthase Kinase 3β (GSK3β), which is inactivated by pAKT (Figure 1). mTORC2-deficient T cells, which do not fully activate AKT, show reduced β-catenin and TCF-1 (85). Nonetheless, most studies implicating TCF-1 in Tfh cell generation have been done in the context of strong Th1-inducing infections (88–90), and how these findings relate to Tfh cells in other contexts remains unknown. Thus, the relationship between PI3K and TCF-1, how they affect Tfh cell differentiation, and involvement in possible feedback loops remain intriguing questions.

## PI3K Pathways in Tfr Cells

A subset of thymic derived T regulatory cells, defined as T follicular regulatory (Tfr) cells, are localized at the T-B cell border and inside the GC area (99), and directly control the activation and differentiation of Tfh and GC B cells, including the development of autoimmunity (100). Tfr cells are phenotypically similar to Tfh cells and express BCL-6 (101–103), yet lack expression of B-cell-helper molecules, such as CD40L, and express inhibitory molecules CTLA-4, GITR, IL-10, and granzymes (104). Although excessive PI3K-mTOR activity is detrimental for induced-Treg cell differentiation (105–107), *Pik3cd*^E1020K/+^ mice show increased Treg and Tfr cells at steady state (16, 26). Indeed, although Tfr cells derive from Tregs, Tfr have different requirements for their differentiation and function. For example, IL-2 is necessary for Treg development and suppressive capability (108), yet prevents Tfr cell differentiation in a BLIMP1-dependent manner, similar to IL-2's effects on Tfh cells (109). Tfr cells also display high mTORC1 activity that promotes differentiation and STAT3 phosphorylation, which induces *Tcf7* and *Bcl6* (110); increased AKT-mTOR activity in Roquin-deficient Treg cells upregulates Tfh cell gene signatures that drive Tfr cell differentiation (111). How PI3K affects ratios of Tfh:Tfr cells, which are important for regulating humoral responses and autoimmunity (104), is less clear; notably, *Pik3cd*^E1020K/+^ mice have parallel increases in both cell populations (16).

## Dysregulated PI3K Pathways in Autoimmunity

In addition to helping antigen-specific humoral responses to vaccination and infection, Tfh cells have been linked to autoimmunity in both animals and humans (10, 112). Correlations between circulating Tfh (cTfh) cells and disease have been reported in systemic lupus erythematosus (SLE) (113), rheumatoid arthritis (RA) and Sjögren's syndrome (112). Similarly, APDS/PASLI patients have high cTfh cells (16), and autoimmune manifestations including autoantibodies, cytopenias and glomerulonephritis (15, 29). In parallel, we found that *Pik3cd*^E1020K/+^ mice develop autoantibodies against a wide range of self-antigens. Indeed, PIDs caused by mutations affecting PI3K signaling cascades, or “immune TOR-opathies,” often display a combination of defective immune-responses and autoimmunity (114); animal models demonstrate that PI3K activity in B cells, T helper and regulatory T cells contributes to autoimmune manifestations (16, 115–121). Additionally, increased PI3K activity has been observed in several autoimmune diseases (121), and inhibitors of PI3Kδ and PI3Kγ are currently being explored in pre-clinical models of RA and SLE, and clinically for psoriasis (NCT02303509) and Sjögren's (NCT02775916) (121, 122).

Recent data suggest that certain autoantibodies cross-react with gut microbiota, supporting links between the microbiome and autoimmunity (123). Interestingly, we found increased local and systemic immune responses against gut commensals in *Pik3cd*^E1020K/+^ mice, with evidence for cross-reactivity between anti-self and anti-bacterial antibodies. Furthermore, autoantibodies could be prevented by systemic antibiotic treatment (16). Such data highlight roles for PI3Kδ in modulating T and B lymphocyte activation, including that induced by the microbiota, which can lead to autoimmunity.

## Concluding Remarks

Together, a growing body of evidence supports a connection between PI3Kδ and Tfh cell differentiation, raising the possibility that altered PI3K pathways may contribute to both immunodeficiency and autoimmunity. Nonetheless, results during viral infection suggest that effects of PI3K on Tfh cell differentiation may be context-dependent and that PI3K may promote multiple effector cell lineages. A recent report demonstrated that treatment of APDS/PASLI patients with leniolisib, a selective PI3Kδ inhibitor, showed promising improvements in cellular dysfunction and lympho-proliferation (124) Notably, selective PI3Kδ inhibition reduced serum IgM *in vivo* (124), while increasing IgG class-switching *in vitro* (16, 27); however, effects on Tfh cells and autoantibodies have not yet been reported. Our results further suggest that evaluation of microbiota composition and systemic responses to gut commensals in APDS/PASLI may provide new opportunities, possibly in association with leniolisib, for managing this and other conditions where Tfh cells and autoantibodies contribute to pathogenesis. Such approaches may also be relevant for autoimmunity induced by checkpoint-blockade therapy, where PI3Kδ-inhibition may provide a selective control of immune responses. Finally, PI3Kδ activation may help improve vaccine responses, although this would have to be carefully assessed. Thus, a more comprehensive understanding of PI3K regulation and signaling in T and B cells is of crucial importance to more effectively improve humoral immune responses while minimizing autoimmunity.

## Methods

### Animal Care and Ethics

Control (C57Bl/6J) and *Pik3cd*^E1020K/+^ mice (16) were maintained and treated under specific pathogen-free (SPF) conditions under protocols reviewed and approved by the NINDS (protocol 1295-12) and NHGRI (protocol G98-3) Animal Care and Use Committees at the NIH.

### LCMV Infection and Flow-Cytometry

Mice were injected intravenously (i.v.) with 2^*^10^5^ plaque-forming units (PFUs) of LCMV Armstrong, kindly provided by Dorian McGavern Lab, grown as previously described (88). Day+7/8 post infection, single cell suspensions were prepared from spleen in MACS buffer (PBS with 2% FBS and 2 μM EDTA). GP66 tetramer [I-A(b) QVYSLIRPNENPAHK PE] was obtained from NIH tetramer facility (Emory University); staining was performed at 37°C for 1 h in RPMI with 10% serum. CXCR5 staining was performed using: CXCR5-purified (2G8, BD Biosciences), followed by Biotin-SP AffiniPure Fab Fragment Goat Anti-Rat IgG (H+L) (Jackson ImmunoResearch), and Streptavidin (BioLegend) as previously described (88). The following antibodies (obtained from BioLegend, BD Biosciences, eBioscience) were incubated with spleen cells for 45/60 min on ice: CD4 (RM4-5), B220 (RA3-6B2), PD-1 (RMP1-30), SLAM (TC15-12F12.2). Intracellular staining of Foxp3 (FJK-16s) was performed using the Foxp3-staining buffer (eBioscience). Cells were gated according to FSC-A/SSC-A, doublet exclusion (SSC-H/SSC-W and FSC-H/FSC-W), live cells (negative for LIVE/DEAD® Fixable Aqua Dead Cell Stain Kit, Life Technologies), followed by gating strategies indicated in figure legend. Flow cytometry was performed on a LSRII (BD Biosciences) and data analyzed using FlowJo 9.9 software (TreeStar).

### Statistical Analysis

Data were analyzed via Prism 6 (GraphPad Software) using non-parametric unpaired Mann-Whitney *U*-test. Graphs show the mean ± SEM. ^*^P < 0.05; ^**^ *P* < 0.01; ^***^ *P* < 0.001. If not indicated, the *P*-values were not significant (>0.05).

## Author Contributions

SP designed and performed experiments, analyzed and interpreted data, wrote the manuscript and prepared the figures. BH and JLC performed experiments, edited the manuscript, and contributed to discussions. DBM contributed essential reagents and advice. PLS conceived the project, wrote the manuscript, and provided overall direction of the study. All authors concur with this submission.

### Conflict of Interest Statement

The authors declare that the research was conducted in the absence of any commercial or financial relationships that could be construed as a potential conflict of interest.
